# Patients with systemic sclerosis and low CD4 numbers after autologous stem cell transplantation have a favorable outcome

**DOI:** 10.1186/s13075-024-03300-1

**Published:** 2024-03-20

**Authors:** Ann-Christin Pecher, Reinhild Klein, Ina Koetter, Marieke Wagner, Wichard Vogel, Stefan Wirths, Claudia Lengerke, Joerg Christoph Henes

**Affiliations:** 1 Department of Hematology, Oncology, Clinical Immunology and Rheumatology, University Hospital Tuebingen, Otfried-Mueller-Strasse 10, 72076 Tuebingen, Germany; 2 Division of Rheumatology and Systemic Inflammatory Rheumatic Diseases, University Hospital Hamburg-Eppendorf and Clinic for Rheumatology and Immunology, Bad Bramstedt, Germany

**Keywords:** Systemic sclerosis, Autoimmunity, Autologous stem cell transplantation

## Abstract

**Background:**

Treatment with high-dose chemotherapy followed by autologous hematopoietic stem cell transplantation (aHSCT) is an intensive treatment option for patients with severe forms of systemic sclerosis (SSc). Even though associated with a high treatment related mortality, the results in this high-risk population are generally favourable. The knowledge on the potential mechanism of action of this therapy and how it can improve patients with SSc is crucial to better select the right patients for aHSCT.

**Methods:**

This is a monocentric retrospective study from Tübingen, Germany, including 32 patients who underwent aHSCT. Peripheral blood samples were analysed for different lymphocyte subsets at various timepoints before and after aHSCT. Patients were divided into responders and non-responders according to the modified Rodnan skin score and lung function test in the three years following aHSCT.

**Results:**

Responders showed significantly lower levels of cluster of differentiation (CD)4 positive T cells in the first months after aHSCT (month 1 and 3), B cells (month 3 and 6 after aHSCT) and natural killer cells (month 1). Mantel-cox test showed a significant deviation of the probability curves, i.e. patients with lower CD4 + T cells and natural killer cells one month and B cells after 3 months after stem cell transplantation had a higher probability to belong to the responder group.

**Conclusions:**

Taken together, this study supports the theory that a profound CD4 + T cell and B cell lymphopenia is important for patients with SSc to achieve a sustained response after aHSCT.

## Introduction

Systemic sclerosis (SSc) is a rare connective tissue disease with a high disease-related mortality, mostly attributed to pulmonary fibrosis or heart involvement [1, 2]. Its complex pathogenesis remains incompletely understood. It is acknowledged, though, that the pathogenesis of SSc is characterized by the following main features: first, vasculopathy, second, inflammation accompanied by the production of auto-antibodies and third, tissue fibrosis through proliferation and differentiation of fibroblasts.

Autologous hematopoietic stem cell transplantation (aHSCT) is considered the most effective treatment option in selected patients with refractory SSc. Since 1996 data on aHSCT in SSc have been published regularly [3–11], which demonstrated the effectiveness of aHSCT in autoimmune diseases, especially with regard to long term progression-free survival, sometimes with dramatic improvements or resolution of skin fibrosis and stabilization or even improvement of pulmonary fibrosis. Even normalizations of pathological capillary changes (nail fold capillaries) have been shown [12, 13], as have histological improvements in skin fibrosis after autologous stem cell transplantation [14, 15]. Nevertheless, besides its proven benefit, aHSCT is a therapy with a risk for treatment related mortality (published reports range from 6 to 12%) and patients have to be selected carefully [6, 8, 10, 11].

Although the results of aHSCT in this patient cohort suggest an overall promising outcome, the mechanism of action, however, is not yet fully understood. Furthermore, some patients do not improve or show deterioration after initial improvement, maybe due to reinfusion of autoreactive cells or autoreactive cells surviving the intensive chemotherapy of the conditioning treatment. It is suggested that the re-transfusion of the stem cells plays a central role in the concept of aHSCT: The return of immunologically undifferentiated cells enables a new “learning phase” and thus the learning of self-tolerance, which is disturbed in autoimmune diseases. CD4 + T cells recover via thymopoiesis and develop a new T cell receptor repertoire and therefore a profound CD4 + T cell lymphopenia has been assumed as a cause of action [16–19]. B cells also emerged in the focus of interest in the pathogenesis [20]. Therefore, the radical depletion of autoreactive T and B cells might be important, whether a patient achieves a (long term) treatment response.

This retrospective study of patients with SSc and aHSCT analyzes the reestablishment of the immune system following aHSCT and whether the different cells of the immune system, especially CD4 + T cells and B cells, develop differently in patients who reach a sustained respond to this therapy in comparison to non-responders.

## Methods

### Patients

This is a retrospective, monocentric study at the Department of Hematology, Oncology, Clinical Immunology, and Rheumatology, University Hospital Tuebingen, Germany. We analysed an older cohort of all patients with systemic sclerosis, who underwent stem cell transplantation from 1997 to 2012 and were not included in any other study. Patients were divided into two groups according to their response (defined in the section treatment outcome): primary and ongoing responders versus (vs.) primary non-responders and patients with relapse in the first three years following aHSCT. We defined this time point, as the SCOT trial [11] showed a sustained response to therapy for those patients who reached the three-year-mark.

This retrospective analysis was approved by the institutional review board of the Eberhard-Karls-University Tuebingen (IRB approval number 348/2016BO2) to be in accordance with the ethical standards and with the Helsinki Declaration.

### Peripheral blood stem cell collection and autologous stem cell transplantation

Peripheral blood stem cells (PBSC) were mobilized by administration of recombinant human G-CSF (from Day 4 until apheresis) after chemotherapy with cyclophosphamide (CYC; 2 × 1–2 g/m² body surface) for 2 consecutive days and collected according to local standards, beginning on day 10. In one patient - due to CYC intolerance -, stem cells were obtained by bone marrow aspiration without previous chemotherapy. We aimed for a cell count > 2 × 10^6^ CD34 + cells/kg bodyweight (BW), and all cells were positively selected via immunomagnetic beads (CliniMACS® CD34 Reagent System, Miltenyi, Bergisch Gladbach, Germany).

Standard pre-transplant conditioning regimen contained of CYC 50 mg/kg bodyweight (day − 5 until day − 2) and T-cell depletion therapy with rabbit antithymocyte globulin (ATG, grafalon®, Neovii Pharmaceuticals AG, Switzerland) in varying doses from 2.5 to 40 mg/kg BW (day − 4 until day − 1), or thiotepa 2 × 5 mg/kg BW (day − 5), ATG 2.5–40 mg/kg BW (day − 4 until day − 1), and CYC 50 mg/kg BW (day − 3 until day − 2) depending on the disease manifestation as described previously [7]. We used melphalan 100 mg/m² body surface area (BSA) in the one patient intolerant to CYC. Frozen-thawed CD34 + positively selected PBSC were transplanted on day 0. There was no application of G-CSF after reinfusion of autologous stem cells in any patient.

### Treatment outcome

We defined an improvement in the skin or lung manifestations as a response to aHSCT. However, in the case of SSc, no disease progression might also be evaluated as a treatment success. As it is very difficult to predict the natural course of the disease, we limited the outcome in the response group to “improvement” to minimize selection bias in this retrospective analysis.

Response to treatment was defined as a 25% improvement of skin sclerosis measured by the modified Rodnan skin score (mRSS) [21] and/or 10% of lung function tests (diffusing capacity for carbon monoxide (DLCO) or forced vital capacity (FVC)) for at least three years after aHSCT. Disease progression and relapse were defined as any worsening of skin fibrosis at two timepoints from the same examiner or decrease of DLCO with new ground-glass pattern as a sign for alveolitis in high resolution computed tomography (HRCT) scans in the three years following transplantation. We did specifically not differentiate between ongoing responders and relapsing patients after three years as this analysis focusses on the reconstitution of the immune system after aHSCT.

### Anti-topoisomerase I levels

Serum levels of anti-topoisomerase I antibody (anti-Scl-70) were measured by Enzyme-linked Immunosorbent Assay (ELISA) as described previously [22], however one patient from this former analysis was excluded as he was treated in a trial protocol and therefore not analyzed in the work presented here.

### Flow cytometry for cell phenotyping

We collected whole blood samples from patients before aHSCT (pre) with varying timepoints before mobilization chemotherapy and on the following timepoints after HSCT (in months): 1, 3, 6, 9, 12, 18, 24, 30, 36, 42, 48, 54, 60. Samples collected after 5 years after aHSCT were summarized (post).

Lymphocyte immunophenotyping of samples was performed with BD FACSCalibur™ using conjugated antibodies from BD Biosciences™: anti-human CD3 FITC (SK7), anti-human CD45 PerCP (2D1), anti-human CD8 PE (SK1), anti-human CD4 APC (SK3), anti-human CD19 APC (2SJ25C1), anti-human CD16 PE (B73.1), anti-human CD56 PE (NCAM16.2) and FITC Mouse IgG1, κ Isotype Control.

### Statistical analysis

For statistical analysis Prism 9 (GraphPad Software) was used. If not otherwise specified, results are presented as mean +/- standard error of the mean. When range is reported, it shows the smallest and largest values. Data were compared using the Student’s t-test. The Mann-Whitney U test was used for data that were not normally distributed. Correlation was evaluated by determination of the Pearson correlation coefficient. Mantel-cox test was used for comparing Kaplan-Meier curves. *P* < 0.05 was considered statistically significant.

## Results

Thirty-two patients were included, of whom 19 patients (58%) were defined as responders, i.e. ongoing response (defined by mRSS and pulmonary performance) for at least three years. Our therapy regimen developed over time, therefore CYC and ATG doses varied in between patients, but were comparable in between groups (Table 1). Also, we observed more male patients (54% versus 26%), a longer disease duration (43 months versus 33 months), and a higher proportion of anti-Scl70 positivity (77% positive versus 63% positive) in the non-Responder group, these differences however were not significant. The reinfused stem cell products were comparable, although the responder group showed a numeric increase of reinfused CD3 + T cells (mean 0.4 CD3 + cells x10^4^/kg versus 0.2 CD3 + cells x10^4^/kg).

**Table 1:** Patients’ characteristics

Characteristic	All patients	Responder	Non Responder	p value
**Demographics**				
Number of patients	32	19	13	
Female [no.[%)]	20 (63)	14 (74)	6 (46)	0.15
Median age (range)	40 (19–57)	39 (19–53)	42 (19–57)	0.51
**Disease characteristics** [mean, (range)]				
Disease duration (months)	37 (3-125)	33 (3–87)	43 (6-125)	0.35
Anti-Scl70 positive (no.[%])	21 (66)	12 (63)	9 (77)	0.47
mRSS	20 (2–35)	20 (3–35)	18 [2–30]	0.52
No. of organs involved Skin [no.[%)] Lung [no.[%)] Gastrointestinal [no.[%)] Heart [no.[%)] Musculoskeletal [no.[%)] Kidney [no.[%)]	3.0 [1–5] 32 (100) 30 (94) 17 (53) 9 [27] 6 [19] 1 [3]	2.8 [1–5] 19 (100) 17 (89) 8 (42) 6 (32) 3 [16] 1 [5]	3.2 [2–5] 13 (100) 13 (100) 9 (69) 3 [22] 3 [22] 0	0.34 > 0.9 0.50 0.17 0.70 0.67 > 0.9
No. of prior therapies	2.0 (0–8)	1.6 (0–4)	2.5 (0–8)	0.12
**Treatment** [mean (range)]				
Reinfused autologous cells Purity [%] CD34 + stem cells x10^6^/kg CD3 + cells x10^4^/kg	89 (41–99) 4.5 (2.3–9.2) 0.3 (0.1–2.2)	89 (46–99) 4.5 (2.3–9.2) 0.4 (0.1–2.2)†	89 (41–99) 4.3 (2.3–6.9) 0.2 (0.1–0.5)	0.97 0.56 0.11
Cyclophosphamide dose Mobilization therapy (g/m²) Conditioning regimen (mg/kg)	3.2 (0–4) 188 (0-200)	3.1 (0–4) 195 (150–200)	3.3 (0–4) 177 (0-200)	0.72 0.22
ATG dose (mg/kg)	34 (2.5–40)	34 (2.5–40)	35 (2.5–40)	0.63
Treatment plan including thiothepa	6 (19%)	4 (21%)	2 (15%)	> 0.9

*Abbreviations*: aHSCT indicates autologous hematopoietic stem cell transplantation; mRSS modified Rodnan skin score; No. number; ATG antithymocyte globulin. †data available for *n* = 16 patients;

**Fig. 1:**
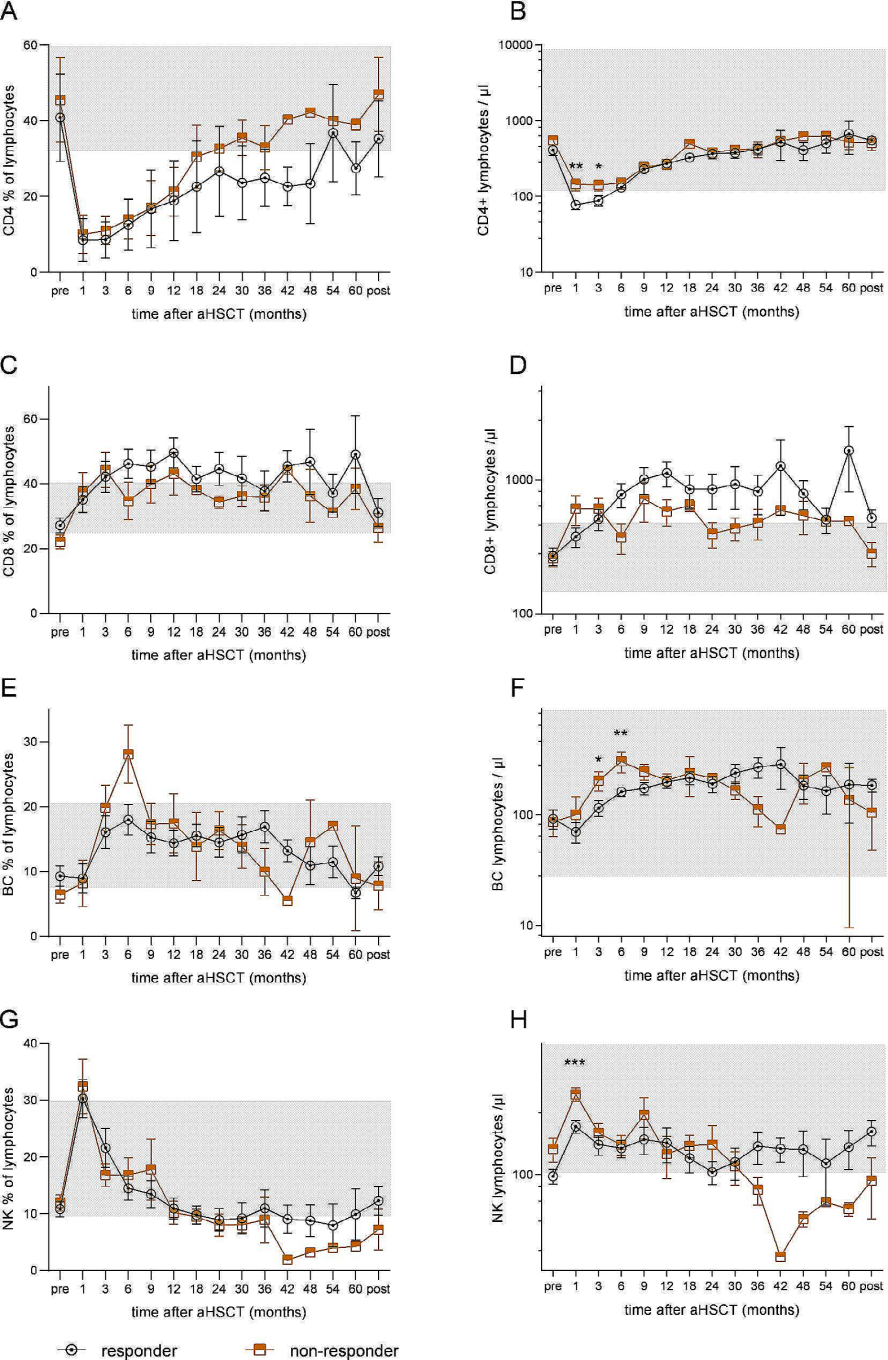
Reconstitution of different lymphocyte subsets after autologous hematopoietic stem cell transplantation (aHSCT). Absolute and percentual cell counts on different timepoints from (A + B) CD4 + T cells (TC), (C + D) CD8 + TC, (E + F) B cells (BC), and (G + H) Natural Killer lymphocytes (NK). Non-responders (circle, black) compared to responders (square, brown) had significantly higher absolute CD4 + TC numbers on month 1 and 3 after aHSCT (77.7/µl vs. 144.6/µl; *p* < 0.01 and 88.9/µl vs. 142.2/µl; *p* < 0.05 respectively), higher absolute BC counts on month 3 and 6 after aHSCT (115.2/µl vs. 203.6/µl; *p* < 0.05 and 161.2/µl vs. 303.2/µl; *p* < 0.01 respectively) and higher absolute NK cells on month 1 after aHSCT (223.4/µl vs. 383.7; *p* < 0.001). The normal values are highlighted in gray. * *p* < 0.05, ** *p* < 0.01

Patients’ samples were analysed via immunofluorescence staining for different lymphocyte subsets at different time points before and after aHSCT: T helper cells (CD4 + TC), cytotoxic T cells (CD8 + TC), Natural killer cells (NK) and B cells (BC). There was no significant difference between groups for any cell types analyzed prior aHSCT. In addition, we observed no significant difference in absolute lymphocyte counts, relative cell counts of different subsets of lymphocytes (i.e. percentage of CD4 + TC, CD8 + TC, BC and NK) and absolute CD8 + TC between groups after aHSCT in the reconstitution of the immune system following aHSCT (Fig. 1A,C,D,E,G).

However, the absolute NK count was significantly lower in the responder group in the first month (223.4/µl vs. 383.7; *p* < 0.001) following aHSCT, as were CD4 + TC counts in the first 3 months after aHSCT (77.7/µl vs. 144.6/µl; *p* < 0.01 and 88.9/µl vs. 142.2/µl; *p* < 0.05 respectively) and BC at the timepoints 3 and 6 months (115.2/µl vs. 203.6/µl; *p* < 0.05 and 161.2/µl vs. 303.2/µl; *p* < 0.01 respectively) after aHSCT (Fig. 1).

We also compared the CD4/CD8 ratio in both groups in the months following aHSCT (data not shown). There was a significant deviance in month 6 (CD4+/CD8 + TC ratio 0.39 vs. 0.70, *p* < 0.05), all other time points did not show a significant difference.

**Fig. 2:**
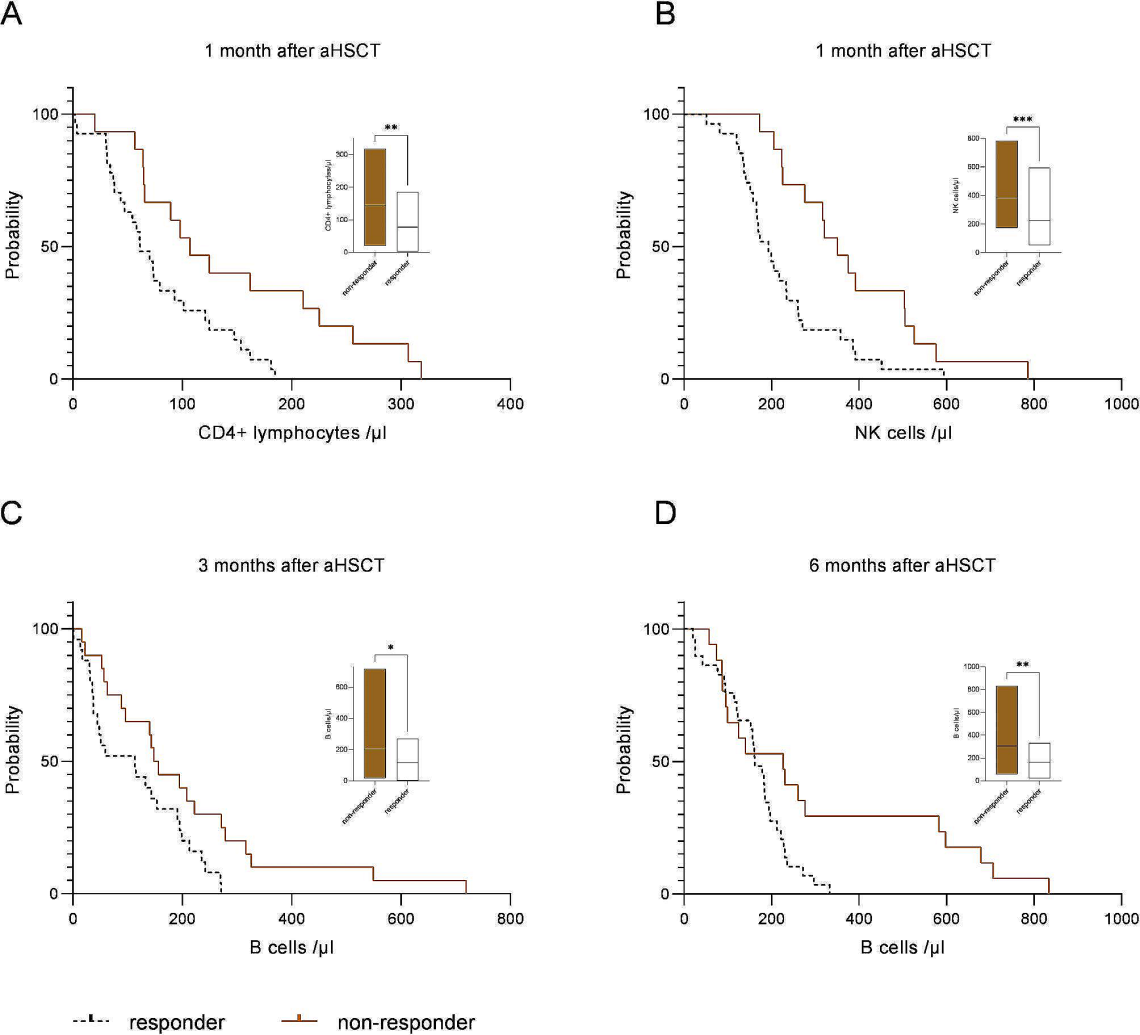
Probability curves in Kaplan-Meier-technique at different timepoints following autologous hematopoietic stem cell transplantation (aHSCT). Area under the curves (AUC) were significantly different comparing responders (dotted line, black) and non-responder group (brown) for (**A**) CD4 + T cells (*p* < 0.01) and (**B**) Natural Killer (NK) cells after 1month (*p* < 0.01) and also for (C + D) B cells (BC) 3 and 6 months after aHSCT (both *p* < 0.05). Small graphs (min to max; line = mean) illustrate the difference between responders (white) and non-responders (brown) at each time point. * *p* < 0.05, ** *p* < 0.01; *** *p* < 0.001

To further investigate the possibility of different lymphocyte subsets to predict the outcome of aHSCT we generated Kaplan-Meier analyses for different time points after aHSCT and different lymphocyte subsets. Figure 2 demonstrates selected curves, where Mantel-cox test showed a significant deviation of the probability curves. One month after aHSCT the probability to belong to the responder group was higher with lower CD4 + TC and NK cell counts (Fig. 2A+B). This was also the case for lower BC counts 3 + 6 months after aHSCT (Fig. 2C + D).

## Discussion

 Herein we analyzed a cohort of 32 patients with SSc receiving high-dose chemotherapy followed by autologous stem cell transplantation with a 5-year follow up regarding the reconstitution of the immune system. To the best of our knowledge, this is the largest cohort done so far on this topic. Furthermore, we included samples over a long-lasting time period before and after aHSCT and also provide data on the individual stem cell product which might influence immune reconstitution and response to therapy. Our analysis suggests that patients with fewer CD4 + TC and NK cells in the first months after aHSCT are significantly more often long-term responders. The lower CD4 + TC number cannot be explained by transfused TC in the stem cell product, as the difference in between groups was not significant, with even a slightly higher number of reinfused TC in the responder group. We did also observe a significant slower reconstitution of B cells (month 3 and 6 after aHSCT) as has been shown by other smaller cohorts with 7 and 10 patients before [18, 23]. Therefore, this study supports the theory, that at least initially, a profound CD4 + and BC lymphopenia is important for patients to achieve a sustained response after aHSCT. The mechanism remains unclear, but it is suggested, that a profound lymphopenia also eradicates the autoreactive clones of B- and T-cells, which might be the drivers of autoimmunity

 The mobilization and conditioning regimens of aHSCT in patients with SSc developed over time. Therefore, the herein analyzed patients were not treated by a standardized therapy protocol regarding the applied chemotherapy and the therapy after aHSCT, as some patient received thiothepa or low-dose glucocorticoids and others not. Especially glucocorticoids and different dosage of ATG (with however similar dosage in both groups) might have had an impact on lymphocyte counts. In addition, it cannot be ruled out that the start of immunosuppressive medication in the case of progressive disease had an effect on lymphocyte counts. However, our results showed low numbers in the responder group, so we think that the re-initiation of immunosuppressive medication can be neglected in this case. We also calculated the proportion of lymphocyte subsets, which showed no significant difference, and the CD4+/CD8 + TC ratio, with a significant lower ratio for responders in month 6 after aHSCT. Nevertheless, after aHSCT the general lymphocyte count is low, therefore percentage values might underestimate the changes seen in different patients during immune reconstitution

 Whereas innate immunity recovers in few months after aHSCT, the adaptive immunity takes 1–2 years to reestablish and some patients suffer from longer-lasting deficits [24]. Most of our patients reached normal lymphocyte values for BC and CD8 + cells after less than 6 months, CD4 + TC however persisted low in the first two years following aHSCT, resulting in an inversed CD4+/CD8 + TC ratio in this time period. In patients with hematological disorders receiving aHSCT, a low BC count is observed in the first months after aHSCT with an increase over 3–18 months and CD4 TC remain low for at least a year or longer, whereas CD8 + TC return sooner, i.e. 3–18 months after aHSCT [24, 25]. Therefore, reconstitution of the lymphocytes is comparable to patients with hematological diseases and other autoimmune diseases

 We registered significantly more male patients in the non-responder group, male gender being a known risk factor for an aggressive disease course [26]. It cannot be excluded that gender might also have an influence on the reconstitution of the immune system after aHSCT, as both, gender and age, have an impact on lymphocyte counts [27]. Furthermore, there is a heterogeneity of the non-responder group, as some of them showed no improvement to aHSCT and some relapsed. The possibility of different pathophysiological mechanisms should be taken in account

 Unfortunately, we are not able to analyze further CD4 + and CD8 + subsets as for example naïve TC and BC, effector TC, memory TC and Th17-/ Th1-/ Th2-subgroups mostly due to the fact, that some samples were collected before 2000 and only few cells were available. Over the past years, it has been demonstrated that these subtypes matter differently in autoimmune diseases and probably have an influence on the chances of improvement in the course of therapy. Other authors could demonstrate that the reconstitutional process is dominated by naïve cells compared to memory cells [28].

 Even though our data underline the importance of an early profound lymphopenia, further analyses of the immune reconstitution and of how profound lymphopenia might influence the outcome of aHSCT are necessary. In particular, the question remains whether profound lymphopenia results from a depletion of an early autoreactive stem cell or the elimination of an abundant number of peripheral autoreactive cells and if a particular subset is of primary importance. This might also have an impact on further conditioning regimen: first, if profound lymphopenia is paramount, “milder” protocols which could be discussed to reduce CYC or other chemotherapy might be less effective and the intensive chemotherapy regimen are necessary despite the side effects and the associated high treatment-related mortality. And second, to better apprehend the significance of more specific therapies as ATG and rituximab or even daratumumab. These therapies might partly substitute intensive chemotherapy regimens if a certain lymphocyte subset proves to be of utmost importance. Consequently, this might result in a less toxic therapy and thus reduce morbidity and mortality. Furthermore, it has been shown that weight-based dosing of ATG shows varying exposure in different individuals and is associate with response to therapy [29]. This may pave the way for not only weight-based but also lymphocyte count- adapted therapy, as has already been attempted in pediatric patients undergoing allogenic stem cell transplantation [30].

## Data Availability

No datasets were generated or analysed during the current study.

## Abbreviations

Anti- Scl 70: Anti- topoisomerase I antibody
aHSCT: Autologous stem cell transplantation
ATG: Antithymocyte globulin
BC: B cells
BSA: Body surface area
BW: Body weight
CD: Cluster of differentiation
DLCO: Diffusing capacity for carbon monoxide
EBMT: European Bone Marrow Transplantation
ELISA: Enzyme-linked Immunosorbent Assay
EULAR: European League Against Rheumatism
HRCT: High resolution computed tomography
mRSS: Modified Rodnan skin score
NK: Natural killer cells
PBSC: Peripheral blood stem cells
SSc: Systemic sclerosis
TC: T cells
TCR: T cell receptor
FVC: Forced vital capacity
vs.: Versus
